# Immersive Virtual Reality for Stroke Rehabilitation: Linking Clinical and Digital Measures of Motor Recovery—A Pilot Study

**DOI:** 10.3390/bioengineering13010059

**Published:** 2026-01-04

**Authors:** Livia-Alexandra Ion, Miruna Ioana Săndulescu, Claudia-Gabriela Potcovaru, Daniela Poenaru, Andrei Doru Comișel, Ștefan Ștefureac, Andrei Cristian Lambru, Alin Moldoveanu, Ana Magdalena Anghel, Delia Cinteză

**Affiliations:** 1 National Institute of Rehabilitation, Physical Medicine and Balneoclimatology, 030079 Bucharest, Romania; 2 Doctoral School, Lucian Blaga University of Sibiu, 550024 Sibiu, Romania; 3 Physical Medicine and Rehabilitation, “Carol Davila” University of Medicine and Pharmacy, 050474 Bucharest, Romania; 4 Computers Department, Faculty of Automatic Control and Computers, National University of Science and Technology Politehnica Bucharest, 060042 Bucharest, Romania; 5 Automatic Control and Applied Informatics Department, Faculty of Automatic Control and Computers, National University of Science and Technology Politehnica Bucharest, 060042 Bucharest, Romania

**Keywords:** stroke rehabilitation, immersive virtual reality, neuroplasticity, motor recovery, feasibility study, usability, data-driven rehabilitation

## Abstract

Background: Immersive virtual reality (VR) has emerged as a promising tool to enhance neuroplasticity, motivation, and engagement during post-stroke motor rehabilitation. However, evidence on its feasibility and data-driven integration into clinical practice remains limited. Objective: This pilot study aimed to evaluate the feasibility, usability, and short-term motor outcomes of an immersive VR-assisted rehabilitation program using the Travee-VR system. Methods: Fourteen adults with post-stroke upper-limb paresis completed a 10-day hybrid rehabilitation program combining conventional therapy with immersive VR sessions. Feasibility and tolerability were assessed through adherence, adverse events, the System Usability Scale (SUS), and the Simulator Sickness Questionnaire (SSQ). Motor outcomes included active and passive range of motion (AROM, PROM) and a derived GAP index (PROM–AROM). Correlations between clinical changes and in-game performance metrics were explored to identify potential digital performance metrics of recovery. Results: All participants completed the program without adverse events. Usability was rated as high (mean SUS = 79 ± 11.3), and cybersickness remained mild (SSQ < 40). Significant improvements were observed in shoulder abduction (+7.3°, *p* < 0.01) and elbow flexion (+5.8°, *p* < 0.05), with moderate-to-large effect sizes. Performance gains in the Fire and Fruits games correlated with clinical improvement in shoulder AROM (ρ = 0.45, *p* = 0.041). Cluster analysis identified distinct responder profiles, reflecting individual variability in neuroplastic adaptation. Conclusions: The Travee-VR system proved feasible, well tolerated, and associated with measurable short-term improvements in upper-limb function. By linking clinical outcomes with real-time kinematic data, this study supports the role of immersive, feedback-driven VR as a catalyst for data-informed neuroplastic recovery. These results lay the groundwork for adaptive, clinic-to-home rehabilitation models integrating clinical and exploratory digital performance metrics.

## 1. Introduction

Stroke remains among the leading causes of long-term adult disability globally, with upper-limb motor deficits affecting approximately 80% of survivors in the acute phase and persisting in more than half of patients beyond six months [1,2]. These impairments, characterized by weakness, spasticity, and loss of coordination, severely limit independence in daily activities and reduce quality of life [3]. Despite advances in acute management, the long-term restoration of upper-limb function remains one of the most challenging goals in neurorehabilitation [4].

Motor recovery after stroke is a complex, multilevel process that involves spontaneous neurobiological repair mechanisms, as well as rehabilitation-induced, experience-dependent plasticity. Early improvements in the acute and subacute phases arise primarily from reperfusion, reduction in perilesional inflammation, and dendritic sprouting, while later gains depend on the adaptive reorganization of residual neural networks. Neuroimaging studies have demonstrated compensatory recruitment of ipsilesional and contralesional premotor and parietal areas, as well as changes in functional connectivity within the corticospinal and cerebellar circuits that support voluntary movement control [5]. These dynamic processes reflect the brain’s ability to reorganize sensorimotor representations in response to task-specific training and feedback.

Beyond the structural lesion itself, post-stroke motor recovery is heavily influenced by cognitive and behavioral factors that shape how patients engage with therapy. Attention, visuospatial processing, motor awareness, and executive function determine the ability to plan, monitor, and adapt motor actions. Deficits in these domains, such as unilateral neglect, can significantly limit the benefits of even intensive physical rehabilitation. Conversely, preserved attentional control and self-awareness facilitate learning and transfer of motor skills to daily activities [6]. This cognitive–motor interaction underlies the emerging concept of “embodied cognition” in neurorehabilitation, where movement and perception mutually reinforce neural adaptation [7].

The temporal dynamics of recovery follow a non-linear trajectory: the steepest improvements occur during the first 3–6 months after stroke, coinciding with maximal neuroplastic potential, while later phases show slower, incremental gains driven by training intensity and motivation. This plateau effect highlights the need for interventions capable of sustaining engagement and sensorimotor stimulation beyond the spontaneous recovery window [8,9]. Immersive virtual reality offers a promising approach in this context. By combining repetitive, goal-directed movement practice with multisensory feedback and motivational reward systems, VR-based rehabilitation can reinforce learning loops, strengthen residual pathways, and maintain long-term participation [10].

Beyond global recovery dynamics, lesion side and associated neurocognitive deficits can substantially influence rehabilitation outcomes. Right-hemispheric lesions are frequently associated with spatial neglect and reduced self-awareness (anosognosia), which can limit active task participation and error correction [11,12,13]. In contrast, left-hemispheric strokes often present with language and comprehension impairments such as aphasia or impaired verbal working memory, which can reduce the ability to follow complex instructions and process verbal feedback [14].

These lateralized patterns highlight the need for adaptive and multimodal feedback mechanisms, such as those provided by immersive virtual environments, which can bypass linguistic barriers and increase engagement through visual and kinesthetic cues. These factors may partially explain the variability in responsiveness to standardized rehabilitation protocols and justify the integration of individualized, feedback-driven digital interventions.

Traditional rehabilitation programs rely on repetitive, task-specific training aimed at inducing neuroplastic reorganization within spared cortical and subcortical networks [15]. However, the intensity and engagement required for optimal recovery are often difficult to maintain in clinical practice due to patient fatigue, limited therapist availability, and motivational decline over time. In recent years, virtual reality (VR)–based rehabilitation has emerged as a promising adjunctive approach, capable of delivering high-intensity, interactive, and feedback-rich environments that promote active participation and sustained motivation [16,17].

### 1.1. Virtual Reality in Post-Stroke Rehabilitation

VR technology enables the simulation of realistic tasks within immersive or semi-immersive digital environments, allowing users to perform repetitive movements while receiving multimodal feedback: visual, auditory, and sometimes haptic [18]. By providing goal-oriented challenges and immediate performance feedback, VR fosters motor learning principles such as repetition, feedback, and variability of practice, which are known to enhance neural reorganization and functional recovery [19].

A growing body of evidence supports the use of VR in stroke rehabilitation. Systematic reviews and meta-analyses have shown that VR interventions can produce comparable or superior motor outcomes to conventional therapy, particularly when combined with standard physiotherapy. Moreover, VR-based therapy enhances patient motivation and adherence, often leading to longer training durations and greater enjoyment compared with traditional exercises [20,21,22].

Nevertheless, despite its advantages, the translation of VR into routine clinical practice remains limited by factors such as hardware cost, cybersickness, limited therapist training, and uncertainty regarding optimal dosing and patient selection. Therefore, assessing the feasibility, usability, and preliminary clinical effects of new VR systems is an essential step before broader implementation.

### 1.2. The Travee-VR System

The Travee-VR system is a newly developed VR-based rehabilitation platform designed to support upper-limb motor recovery through a set of interactive games and exercises targeting shoulder, elbow, and wrist movements. The system provides quantitative performance metrics (e.g., accuracy, number of targets, reaction time), allowing continuous tracking of patient progress. Travee integrates task-oriented exercises emphasizing large-amplitude and coordinated upper limb movements that are often limited after stroke but critical for daily living activities [23].

Unlike many commercially available systems adapted from entertainment devices (e.g., Wii, Kinect), Travee was purpose-built for neurorehabilitation, ensuring ergonomic positioning, safe movement amplitude, and adjustable task complexity according to the patient’s functional level. Its design allows therapists to monitor adherence and performance remotely, thus enabling personalized progression over time [24].

### 1.3. Rationale and Objectives

Expanding upon previous clinical research on post-stroke upper-limb recovery, the present study continues the exploration of the Travee Experience as part of a hybrid rehabilitation model integrating conventional and technology-assisted therapy. This observational pilot study aims to assess the feasibility, clinical applicability, and short-term motor outcomes of immersive VR-assisted rehabilitation in individuals with residual post-stroke hemiparesis.

The primary objective was to evaluate feasibility, safety, and user acceptance, operationalized through recruitment and retention rates, session completion, absence of adverse events, and usability/tolerability scores using the System Usability Scale (SUS) and Simulator Sickness Questionnaire (SSQ).

The secondary objective was to estimate short-term motor improvements following a 10-day combined rehabilitation program, based on changes in active and passive range of motion (AROM, PROM) and the GAP index across major upper-limb joints.

The exploratory objective was to investigate the relationship between in-game performance metrics derived from the Travee system and clinical outcomes, aiming to identify potential exploratory digital performance metrics and response clusters that could guide future personalized, adaptive rehabilitation protocols.

### 1.4. Significance of the Study

This study provides an early step toward validating an integrated VR rehabilitation framework that combines clinical and digital outcomes. By capturing both traditional range-of-motion changes and in-game performance data, Travee offers a multidimensional view of recovery that may bridge the gap between clinical assessment and ecological, real-time functional behavior.

Moreover, this work contributes to the growing understanding of individual variability in VR response, which remains a major challenge in post-stroke rehabilitation research [25]. Identifying responders and tailoring VR content to patient-specific needs could enhance treatment precision, efficiency, and long-term adherence.

Ultimately, the findings of this pilot study may inform future multimodal neurorehabilitation strategies, integrating VR with sensorimotor stimulation techniques such as focal vibration or robotic assistance to maximize neural recovery through synergistic mechanisms.

This study therefore bridges technological innovation with clinical feasibility, providing early evidence toward data-informed neurorehabilitation.

## 2. Materials and Methods

### 2.1. Study Design, Objectives and Ethical Approval

This was a prospective, single-center, observational pilot study conducted at the National Institute of Rehabilitation, Physical Medicine, and Balneoclimatology, Bucharest, Romania, between December 2024 and August 2025.

The study followed a pre–post design without a control group, as the primary objective was to assess system feasibility and user tolerance rather than efficacy comparison.

The specific objectives were to:

1. Determine feasibility through adherence and session completion;
2. Assess usability and tolerability using standardized questionnaires (SUS and SSQ);
3. Evaluate pre–post changes in upper-limb motor function (AROM, PROM);
4. Characterize VR performance trajectories across games;
5. Explore correlations between VR and clinical outcomes.

All participants provided written informed consent prior to inclusion. The study protocol was approved by the institutional Ethics Committee of the National Institute of Rehabilitation, Physical Medicine, and Balneology (INRMFB, Bucharest) with the registration number code NRCRT6/18.09.2023 and conducted in accordance with the principles of the Declaration of Helsinki.

### 2.2. Participants

**Figure 1:**
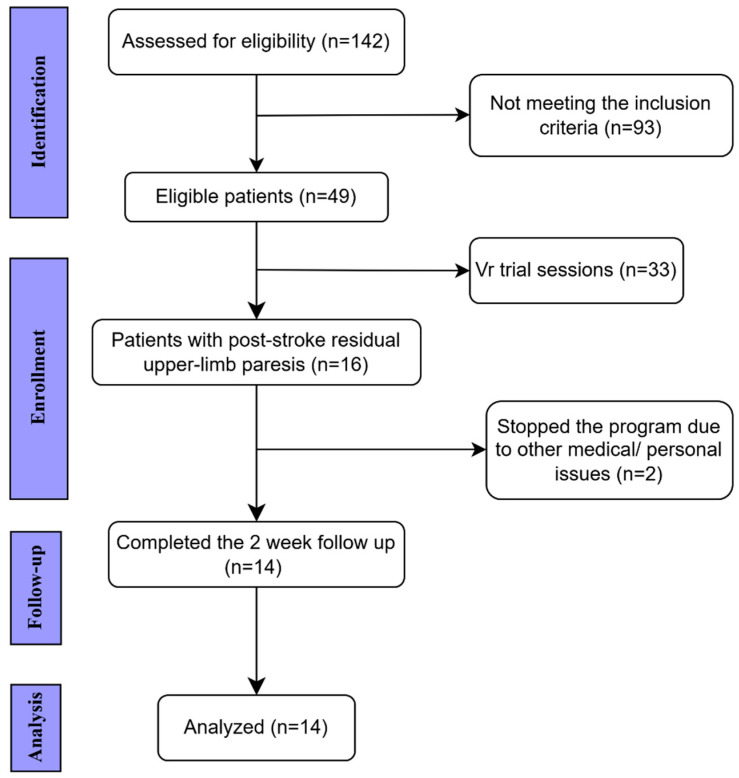
STROBE flow chart of the study. Of the 142 patients screened for eligibility, 49 met the inclusion criteria. After initial VR trial sessions, 16 patients with post-stroke upper-limb paresis were enrolled, of whom 14 completed the 2-week intervention and were included in the final analysis. STROBE: STrengthening the Reporting of OBservational studies in Epidemiology.

We analyzed a convenience sample of 14 adults with post-stroke residual upper-limb paresis who participated in a 10-day inpatient rehabilitation program integrating conventional therapy with immersive virtual reality (VR) training. The participant selection flow is summarized in Figure 1.

#### 2.2.1. Inclusion Criteria

1. Ischemic or hemorrhagic stroke confirmed by CT/MRI;
2. Upper-limb paresis with residual voluntary movement sufficient to interact with the VR interface;
3. No cognitive deficits as assessed by Mini-Mental State Examination (MMSE ≥ 25);
4. Cooperative behavior and ability to understand instructions during VR tasks;
5. Time from stroke onset ≥ 2 months;
6. Age < 80 years and >18 years.

#### 2.2.2. Exclusion Criteria

1. Other neurological conditions causing upper-limb paresis;
2. Unstable cardiovascular status or uncontrolled hypertension;
3. MMSE ≤ 25 or severe cognitive/psychiatric disorders limiting participation;
4. Epilepsy or seizure history;
5. Severe visual or severe vestibular disorders, chronic vertigo, or other conditions associated with intolerance to visual motion (e.g., migraine with aura, Ménière’s disease);
6. Inability to attend the full 10-day rehabilitation program.

These criteria were selected to ensure participants could safely engage in immersive VR tasks requiring arm elevation and visual feedback.

From each patient’s medical record, demographic and clinical data were collected, including stroke type and chronicity (time since onset), comorbidities, smoking and alcohol habits, and hand dominance.

Participants were in the subacute (≥2 months) or chronic (>6 months) phase of recovery and presented mild-to-moderate upper-limb motor deficits (SIAS 3–4) with preserved cognitive function (MMSE ≥ 25). This threshold ensured sufficient cognitive capacity for understanding instructions, maintaining attention, and performing feedback-driven tasks within the VR environment.

Lesion side (right, left, or bilateral) was documented for all participants based on neuroimaging reports. In this cohort, 11 patients had right-hemispheric lesions, 2 had left-hemispheric lesions, and 1 presented bilateral involvement. This information was used to describe cohort characteristics but not for subgroup analysis due to the small sample size. This limited degree of clinical variability was intentionally accepted to reflect real-world rehabilitation populations and was considered appropriate for this pilot feasibility study focused on usability, safety, and preliminary motor outcomes.

### 2.3. Timing of Assessments

Clinical evaluations were performed at two time points: T0 (baseline, pre-intervention) and T1 (post-intervention), immediately after completion of the 10-day rehabilitation program.

The System Usability Scale (SUS) was administered once, at the end of the program, to capture overall user acceptance.

The Simulator Sickness Questionnaire (SSQ) was completed before and after each VR session, and session-level scores were averaged across the 10-day period for analysis.

VR performance metrics were automatically recorded throughout all training sessions, and quantitative analyses were based on the comparison between the first three and last three sessions, reflecting within-program learning and adaptation.

No long-term follow-up was conducted, consistent with the pilot nature of the study, which focused on short-term feasibility, usability, and immediate motor response.

### 2.4. Rehabilitation Protocol

All patients completed a 10-day integrated rehabilitation program combining conventional therapy (CnvT) with immersive VR-assisted training (Travee-VR). Each daily session included approximately 120 min of CnvT and 30 min of Travee-based exercises, supervised by rehabilitation doctors and therapists. The 10-session duration was chosen to reflect standard inpatient rehabilitation cycles and to assess short-term motor adaptability.

#### 2.4.1. Overall Structure and Duration

The 10-day rehabilitation schedule reflected the standard inpatient cycle currently applied in Romanian neurorehabilitation centers, following the framework of the Romanian National Health Insurance House (CNAS), which allocates rehabilitation services in 10-day cycles. This design ensured alignment with real-world clinical practice. The daily therapy dose of 150 min (120 min of conventional therapy and 30 min of immersive VR) was consistent with institutional protocols for moderate post-stroke patients undergoing intensive recovery phases. All participants were in the subacute or chronic stage of recovery, clinically stable, and screened for adequate cognitive and physical endurance (MMSE ≥ 25, no cardiopulmonary instability) to safely tolerate the program. Sessions were continuously supervised and included short rest intervals to prevent fatigue. This structure is consistent with prior evidence demonstrating the feasibility and safety of 30–40 min immersive VR sessions integrated into intensive rehabilitation programs [26,27,28].

#### 2.4.2. Conventional Therapy Component

The conventional component (CnvT) included strength and stretching exercises, passive and active range of motion training, balance and coordination tasks, occupational therapy when indicated. Adjunctive physical modalities (e.g., electrostimulation, ultrasound, massage, splints, or orthoses) were applied as clinically required. All patients followed a standardized protocol adapted to functional capacity.

#### 2.4.3. VR System and Setup

VR-assisted training was performed using the Travee system (Romania), an immersive virtual reality platform for upper-limb neurorehabilitation, integrating visual, auditory, and motion-tracking feedback [24,29]. The system uses a Meta Quest 2 headset (Meta Platforms, Inc., Menlo Park, CA, USA), enabling real-time upper-limb tracking from wrist to fingers without the need for handheld controllers. Movement accuracy and amplitude were visually augmented in the virtual environment, while auditory feedback reinforced correct task performance.

#### 2.4.4. Individualization of Task Difficulty

The Travee-VR intervention allowed for controlled, physician-guided individualization of therapeutic parameters while maintaining a standardized clinical framework. At baseline, each patient underwent a comprehensive assessment conducted by the attending rehabilitation physician, including evaluation of active range of motion, muscle strength, coordination, and endurance. These findings determined the initial configuration of task difficulty, movement amplitude, and response speed within the VR environment. Throughout the 10-day program, session performance was continuously recorded and reviewed by the physician, who adjusted task parameters, such as the number of targets, range of movement, or timing, according to predefined progression rules in the protocol. Adjustments typically involved 5–10% increments to maintain optimal challenge while avoiding fatigue. Progression was introduced only when the patient completed at least 80% of targets without signs of fatigue, pain, or coordination loss. All sessions were conducted under direct medical supervision to ensure patient safety and methodological consistency. Before the first VR-based rehabilitation session, all participants underwent a brief familiarization and training session (approximately 10 min) under medical supervision. This session aimed to ensure adequate understanding of the virtual environment, task requirements, and interaction modalities, as well as to promote comfort and confidence prior to initiating the active intervention protocol.

#### 2.4.5. VR Task Selection

In this pilot study, seven of the available games were selected based on their targeted movement patterns and clinical relevance for post-stroke motor recovery:

- Rocket and Fires: large-amplitude shoulder abduction and flexion;
- Cooking: internal/external rotation control;
- Fruits to Basket: elbow flexion/extension and forearm supination/pronation;
- Road: isolated forearm rotation with sustained trajectory tracking;
- Piano and Diorama: distal wrist and finger extension with visuomotor precision.

#### 2.4.6. VR Performance Metrics

Quantitative performance data were automatically recorded by the Travee-VR platform. High-frequency kinematic data (60 Hz) were recorded by the VR system’s embedded motion sensors. These data were automatically processed to extract temporal and spatial performance metrics, serving as objective indicators of motor learning and task efficiency.

Although multiple in-game parameters (accuracy, reaction time, completion rate, and score) are continuously tracked by the system, for this study, only the most representative metric for each game was extracted for statistical analysis, the one best reflecting task performance and upper-limb engagement.

The selected parameters are summarized in Table 1, together with the corresponding movement patterns targeted by each game. Such metrics are increasingly recognized as potential digital performance metrics in data-driven neurorehabilitation

#### 2.4.7. Safety Monitoring and Cybersickness Assessment

Safety monitoring was performed throughout the 10-day intervention according to institutional procedures and predefined criteria. Adverse events were defined as any new or worsened neurological, musculoskeletal, cardiovascular, or sensory symptom (e.g., pain, dizziness, nausea, fatigue, or visual discomfort) occurring during or shortly after a therapy session. All sessions were supervised by the attending rehabilitation physician, who observed patient responses in real time and recorded any symptoms in the daily log. Participants were also encouraged to self-report perceived discomfort using a brief checklist adapted from the Simulator Sickness Questionnaire (SSQ).

No clinically significant adverse events occurred. Minor transient symptoms, including brief dizziness or visual fatigue, were reported by a few participants but resolved spontaneously, required no medical intervention, and did not interfere with session completion or adherence to the program.

### 2.5. Outcome Measures

Following the completion of the VR intervention sessions, several standardized clinical and kinematic outcome measures were used to evaluate upper-limb motor recovery, muscle tone, usability, and cognitive status.

#### 2.5.1. Motor Function

Active and passive range of motion (AROM and PROM) were assessed at the shoulder, elbow, forearm, and wrist joints using a manual universal goniometer with 1° precision. For each movement: shoulder abduction and flexion, elbow flexion, forearm pronation–supination, and wrist extension, the starting and end positions were carefully aligned with the goniometer’s stationary and movable arms to ensure reproducibility [30]. Each measurement was repeated twice, and the mean value was used for analysis to minimize inter-rater variability.

AROM represented the arc of motion produced voluntarily by the patient without external assistance, while PROM was defined as the range obtained through gentle external facilitation by the therapist, without pain or compensatory movement. Both were expressed in degrees (°) [30].

Additionally, the difference between passive and active range of motion (PROM–AROM) was calculated for each joint as an indicator of how much of the available biomechanical range could be voluntarily recruited. A smaller PROM–AROM difference (GAP index) was interpreted as reflecting improved neuromotor control and more efficient activation within the available joint range. This approach is supported by clinical evidence showing that functional limitations after stroke are more closely related to reduced active, rather than passive, mobility, indicating that a smaller AROM–PROM disparity reflects enhanced neural drive rather than biomechanical restriction [31].

#### 2.5.2. Muscle Tone Assessment

Muscle tone was assessed for major proximal and distal groups (shoulder abductors, elbow flexors, pronators, wrist/finger flexors) using the Modified Ashworth Scale (MAS) [32]. Two trained evaluators independently scored each assessment; disagreements were resolved by consensus. Although spasticity was not a primary endpoint, it was included descriptively to contextualize functional recovery patterns.

#### 2.5.3. Cognitive Screening

Cognitive eligibility was ensured using the Mini-Mental State Examination (MMSE) [33]. A cutoff of ≥25 points was required for inclusion, reflecting adequate cognitive ability to understand VR instructions and actively engage in the training process.

#### 2.5.4. Usability and Tolerability

System feasibility and user experience were evaluated using two validated self-report instruments: the System Usability Scale (SUS) and the Simulator Sickness Questionnaire (SSQ).

The SUS is a 10-item questionnaire widely adopted in digital health research to assess perceived usability, efficiency, and satisfaction with technology-based interventions [34]. It was administered once, at the end of the 10-day program, to capture overall user acceptance and interface clarity. Scores range from 0 to 100, with values ≥ 70 indicating good to excellent usability.

The SSQ quantifies discomfort related to virtual reality exposure, including nausea, dizziness, and oculomotor strain [35]. It was administered before and after each VR session, and daily scores were averaged across sessions to account for intra-individual variability. Scores < 40 are generally interpreted as mild symptoms, consistent with good tolerability.

Together, these instruments provided complementary insights into the feasibility of the Travee-VR system, balancing subjective usability with physiological comfort during repeated immersive exposure

### 2.6. Statistical Analysis

Statistical analyses were performed using Microsoft Excel (Office 365) for data organization and visualization, and IBM SPSS Statistics, version 28.0 (IBM Corp., Armonk, NY, USA) for statistical computations.

Continuous variables were first assessed for normality using both the Kolmogorov–Smirnov and Shapiro–Wilk tests. Normally distributed data were expressed as mean ± standard deviation (SD), whereas non-normally distributed data were summarized as median and interquartile range (IQR). Categorical variables were presented as frequencies and percentages. Given the small sample size and the exploratory nature of the study, non-parametric tests were applied. The Wilcoxon signed-rank test was used to evaluate within-subject differences between baseline (T0) and post-intervention (T1). Where appropriate, Spearman’s rank correlation coefficient (ρ) was calculated to explore associations between clinical measures and VR performance metrics. No missing data imputation was required, as all participants completed both T0 and T1 assessments. To better capture individual variability in rehabilitation response, an exploratory cluster analysis was performed on the normalized ΔAROM and VR performance data. Clusters were generated using k-means partitioning, guided by silhouette and elbow criteria, resulting in three response profiles: good, moderate, and poor responders. This unsupervised approach allowed qualitative grouping of patients based on multidimensional performance patterns rather than single-outcome thresholds. K-means clustering was conducted on normalized variables using Euclidean distance, with the elbow method applied to determine the optimal number of clusters.

For non-parametric comparisons, effect sizes were calculated as *r = Z/√N*, to facilitate interpretation of the magnitude of observed effects. Given the small sample and potential deviations from normality, nonparametric statistics were prioritized to ensure robustness against outliers.

A two-tailed *p*-value < 0.05 was considered statistically significant. Descriptive statistics and visual analytics (boxplots and trajectory plots) were used to illustrate pre–post differences and interindividual trends. Given the pilot nature of the study, all analyses were considered exploratory, aimed at identifying trends and informing future confirmatory research.

Given the limited sample size inherent to a pilot feasibility study, statistical inferences were interpreted with caution. The applied nonparametric and exploratory analyses were considered appropriate for identifying consistent within-subject trends and informing hypotheses for future larger-scale trials.

### 2.7. Data Management and Confidentiality

All data were anonymized before analysis. VR performance metrics and clinical data were stored on encrypted institutional servers compliant with EU GDPR (2016/679) regulations.

Only authorized research personnel had access to the study database.

### 2.8. Summary of Methodological Rationale

This design prioritizes feasibility and exploratory insight over efficacy testing. The combination of standardized clinical measurements with high-resolution VR data allows a multidimensional understanding of motor progress.

The use of objective, automatically captured performance metrics complements traditional assessments and provides a foundation for future development of digital performance metrics in neurorehabilitation research [36].

## 3. Results

A total of 14 patients completed the study (9 males, 5 females), with a mean age of 55.3 ± 8.5 years (range 43–73 years). The majority of strokes were ischemic (11 cases, 78.6%), while 3 cases (21.4%) were hemorrhagic. Lesion distribution showed a predominance of left hemisphere involvement (*n* = 11, 78.6%), followed by right hemisphere (*n* = 2, 14.3%) and one case with bilateral MCA territory infarcts (*n* = 1, 7.1%).

The mean time from stroke onset to enrollment was 21.8 ± 32.2 months, with a median of 9.0 months (IQR 4.2–18.0), reflecting the heterogeneity of the sample and the inclusion of both subacute and chronic patients. Baseline upper limb motor function, assessed with the SIAS U/L Proximal, yielded a mean score of 3.4 ± 0.7, indicating mild to moderate proximal impairment, with sufficient residual movement to engage in VR-based rehabilitation.

Demographic and baseline clinical characteristics of the participants are summarized in Table 2.

Most patients presented with common vascular risk factors. Hypertension and dyslipidemia were the most frequent, each affecting 13 patients (92.9%). Type 2 diabetes mellitus was present in 4 patients (28.6%), while smoking was reported in 7 patients (50.0%) and alcohol consumption in 3 patients (21.4%). These comorbidities are consistent with those typically reported in post-stroke populations [37,38].

As shown in Table 3, participants reported high usability of the Travee VR system, with a mean SUS score of 79.0 ± 11.3 (median 80.5, IQR 69.3–87.0), consistent with the ‘good–excellent’ usability range. SSQ scores increased numerically from baseline (26.4 ± 11.6, median 24.3, IQR 15.9–32.7) to post-intervention (35.5 ± 14.4, median 37.3, IQR 25.7–48.0), but this difference was not statistically significant (Wilcoxon signed-rank test, *p* = 0.135). Importantly, no participant discontinued the intervention due to intolerance, suggesting that VR-based rehabilitation was overall feasible and well accepted. Beyond subjective tolerability, clinical safety was continuously monitored throughout the intervention.

Safety and tolerability:

All participants completed the full 10-day program without interruptions or withdrawals related to adverse symptoms. No clinically significant adverse events were observed. Minor transient symptoms, such as brief dizziness or visual fatigue, were occasionally reported but resolved spontaneously within minutes, required no medical intervention, and did not interfere with session completion or adherence.

These mild and transient symptoms are consistent with previously reported tolerability profiles of immersive VR in post-stroke rehabilitation and confirm that the system was overall safe and well tolerated.

As summarized in Table 4, participants demonstrated significant within-subject improvements in several upper-limb movements following rehabilitation with the Travee-Vr system. We focused on shoulder, elbow, and wrist movements as these were the most functionally relevant and consistently available across all patients

Shoulder abduction AROM increased from 98.6 ± 19.0° to 102.3 ± 18.3° (*p* = 0.0097), while PROM improved from 120.6 ± 17.0° to 124.8 ± 15.4° (*p* = 0.0074). Similarly, shoulder flexion AROM improved significantly from 101.1 ± 31.9° to 104.4 ± 30.9° (*p* = 0.0065).

At the elbow, flexion AROM increased from 135.3 ± 16.2° to 137.6 ± 14.8° (*p* = 0.0020), with parallel gains in PROM (137.4 ± 14.9° to 141.1 ± 10.9°, *p* = 0.0032).

Shoulder external rotation AROM also showed significant gains (41.1 ± 28.1° to 45.3 ± 26.3°, *p* = 0.0045). At the wrist, extension AROM improved from 32.9 ± 22.3° to 35.2 ± 21.4° (*p* = 0.0176), with PROM also increasing (54.4 ± 18.2° to 56.3 ± 17.1°, *p* = 0.0158).

For forearm pronation, AROM remained stable (80.1 ± 3.5° to 80.3 ± 3.6°, n.s.).

Effect size analysis confirmed that shoulder abduction showed a large effect (*r* = 0.61), while elbow flexion demonstrated a moderate effect (*r* = 0.46), supporting the clinical relevance of these changes.

In addition, the PROM–AROM difference (GAP index) showed a slight reduction, although not statistically significant, suggesting a trend toward improved voluntary recruitment within the available biomechanical range

Overall, the most consistent and statistically significant improvements were observed at the shoulder and elbow, reflecting enhanced proximal mobility. Distal gains (wrist extension) were present but of smaller magnitude, while pronation remained largely unchanged. This pattern aligns with the design of the VR exercises, which emphasize large-amplitude reaching and positioning tasks.

It should be noted that most participants presented with varying degrees of upper-limb spasticity, as commonly observed in the chronic post-stroke phase. Several patients received botulinum toxin injections as part of their standard care pathway. However, these interventions were not systematically recorded or analyzed in the present study, as the primary objective was to assess feasibility and motor outcomes associated with VR-based rehabilitation using Travee.

Baseline (T0) clinical conditions of the participants, including initial active and passive range of motion (AROM, PROM) and GAP indices across upper-limb joints, are presented in Table 4. These baseline data correspond to the ‘Pre’ column and were used as reference values for the subsequent analyses.

Given the exploratory nature of this pilot study and the heterogeneity of post-stroke recovery, we complemented the cohort-level analysis with subgroup clustering and representative case descriptions.

**Figure 2:**
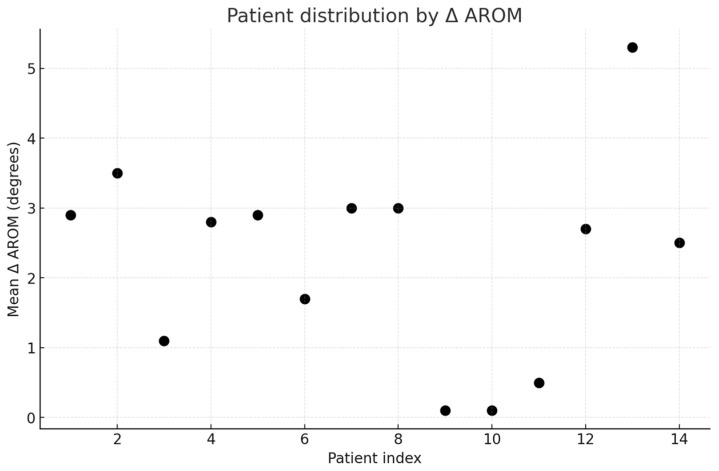
Distribution of patients according to mean Δ AROM (degrees). Patients were stratified into three clusters: poor responders (*n* = 5), moderate responders (*n* = 8), and good responders (*n* = 1).

Exploratory analysis confirmed heterogeneity of clinical responses. Based on mean Δ AROM, patients were divided into poor (*n* = 5), moderate (*n* = 8), and good responders (*n* = 1) (Figure 2), One representative case from each subgroup was selected for detailed description, highlighting the variability of outcomes.

Good responder

Patient #13, a 50 year-old male with a left-sided ischemic stroke in the right MCA territory (14 months post-onset, SIAS UL = 2), exemplified the “good responder” cluster. This patient demonstrated the largest mean Δ AROM (+5.3° across joints), with clear improvements in both shoulder abduction and elbow flexion. PROM values also increased, reducing the passive–active gap, suggesting true motor recovery rather than passive facilitation.

Moderate responder

Patient #12, a 58-year-old male, 48 months after a right MCA territory infarct (left hemiparesis, SIAS UL = 3), was representative of the moderate cluster. His changes were close to the cohort median, with modest but consistent gains in shoulder flexion (+4°) and wrist extension (+3°), while PROM remained stable. This profile illustrates the “average” outcome in our cohort, with measurable but limited recovery potential in the chronic stage.

Poor responder

Patient #9, a 48-year-old male with a right hemispheric hemorrhage (right hemiparesis, 2 months post-stroke, SIAS UL = 4), exemplified the poor responder group. Despite early subacute timing, this patient showed negligible changes in AROM (Δ +0.1° on average), with stable PROM and persistent wide AROM–PROM gaps. This trajectory highlights the heterogeneity of responses, where favorable demographic or clinical factors do not always translate into measurable motor gains.

As presented in Table 5, VR game metrics extracted from the Travee platform revealed heterogeneous performance trajectories across the cohort. The largest and most consistent improvement was observed in the Fire game, where participants extinguished significantly more targets during the last three sessions compared to the initial three (Δ = +36.4, *p* = 0.004). A significant gain was also detected in the Fruits game, with patients catching an average of 6.8 more items by the end of training (*p* = 0.034). Road checkpoints showed a modest nonsignificant increase (Δ = +3.4, *p* = 0.16), while Rocket performance declined slightly, without reaching statistical significance (Δ = −6.0, *p* = 0.31). Diorama completion remained stable throughout, suggesting limited sensitivity of this task to short-term change. Piano and Cooking were played by most participants but did not yield quantitative metrics in this version of the platform.

Importantly, these patterns underscore the heterogeneity of VR learning: while Fire and Fruits captured clear training effects, other games displayed ceiling effects (Road, Diorama) or variable engagement (Rocket). Furthermore, qualitative inspection of individual trajectories revealed dissociations between clinical and VR-based improvements, suggesting that game metrics may capture distinct dimensions of motor performance beyond conventional ROM measures

**Figure 3:**
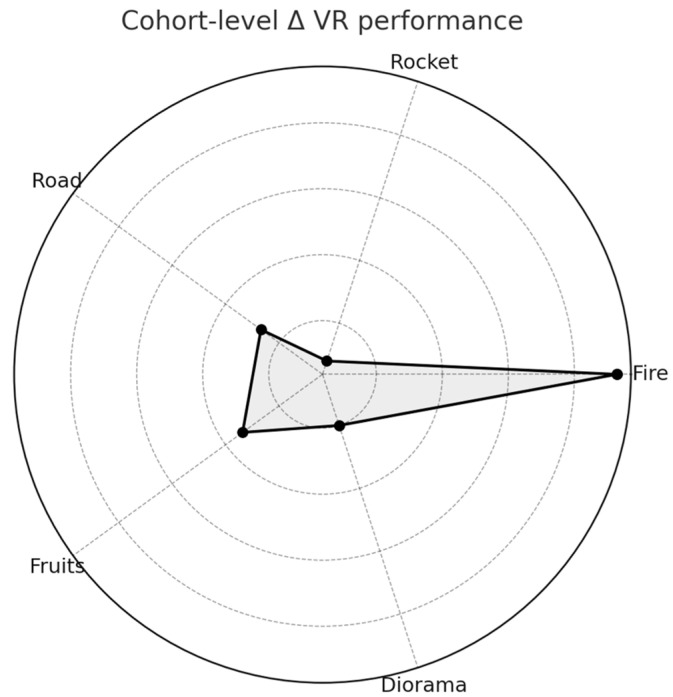
Cohort-level changes in VR performance across Travee games. Radar plot depicting mean change (Δ last–first 3 sessions) across the five Travee games with quantitative metrics (Fire, Rocket, Road, Fruits, Diorama). Fire and Fruits showed the most pronounced improvements, Road showed modest change, Rocket declined slightly, and Diorama remained stable.

At the cohort level, VR performance improved most in Fire and Fruits, while Road showed modest change, Rocket declined slightly, and Diorama remained stable (Figure 3).

**Figure 4:**
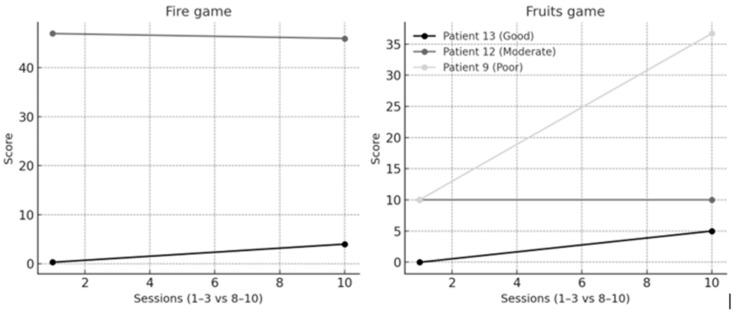
Individual trajectories in VR games. Line plots show Fire and Fruits performance for the three representative patients (good, moderate, poor responders, based on Δ AROM). Patient #13 (good) improved consistently in both games, Patient #12 (moderate) remained stable, while Patient #9 (poor) showed limited clinical gains but clear VR learning in Fruits.

Individual VR trajectories of the three representative patients are presented in Figure 4. Patient #13 (good responder) showed parallel improvements in both Fire and Fruits, consistent with his clinical gains. Patient #12 (moderate responder) remained stable in both games, mirroring modest clinical progress. In contrast, Patient #9 (poor responder) displayed negligible clinical change but demonstrated clear improvement in Fruits, highlighting a dissociation between ROM and VR metrics.

In addition to these representative cases, we identified the top three ‘VR responders’ based on a composite performance score across multiple games. Their results are summarized in Table 6.

Exploratory analysis identified a subgroup of “VR responders”, defined as patients in the top tertile of composite VR scores. Table 6 summarizes the three highest performers. Patient #4 showed consistent improvements across Fire (+55 extinguished targets), Road (+55.6 checkpoints), and Fruits (+18 items), resulting in the strongest composite VR score. Patient #3 demonstrated the largest single-game improvement (Fire +123), while Patient #5 improved across several games, particularly Fire (+106) and Rocket (+28). Notably, these VR gains did not always parallel clinical changes, underscoring the potential of VR metrics to capture distinct aspects of motor learning.

To further explore the relationship between VR performance and conventional clinical outcomes, we examined correlations between game-specific improvements and joint-specific Δ AROM (Table 7).

Exploratory correlations between VR performance and joint-specific clinical outcomes are summarized in Table 7. Fire performance showed a moderate positive correlation with shoulder abduction Δ AROM (ρ = 0.45, *p* = 0.041). No other associations reached statistical significance.

In summary, the program was feasible, safe, and associated with moderate-to-large proximal motor improvements, paralleled by selective gains in VR performance metrics.

## 4. Discussion

Recent advances in post-stroke neurorehabilitation have increasingly integrated technology-assisted modalities such as robotics, telerehabilitation, and virtual reality (VR), aiming to enhance task intensity, patient engagement, and neuroplasticity [20,39]. Previous trials using non-immersive or semi-immersive systems (e.g., Kinect, Wii, or screen-based platforms) have shown that VR-based therapy can achieve comparable or even superior outcomes to conventional therapy when matched for training duration and repetition intensity [18,27].

Fully immersive systems, however, offer higher sensory fidelity and real-time motor feedback, promoting a more natural coupling between visual, proprioceptive, and vestibular inputs. These features may facilitate greater cortical activation and adaptive motor learning compared with traditional or semi-immersive setups [18]. Moreover, hybrid interventions that combine immersive VR with established techniques such as mirror therapy have demonstrated additional benefits in upper-limb function and engagement [40].

In this context, the Travee-VR system represents an evolution of immersive, clinician-supervised rehabilitation tools, designed to combine quantitative digital performance metrics with standardized clinical outcomes. This hybrid, feedback-rich model addresses some of the main limitations of conventional therapy—such as reduced engagement and therapist dependence—while preserving the principles of task specificity and motor repetition fundamental to neuroplastic reorganization [41].

The current pilot study demonstrates that immersive virtual reality (VR)–assisted rehabilitation using the Travee-VR system, combined with conventional therapy, is both feasible and associated with improvements in upper-limb motor performance in individuals with post-stroke hemiparesis. All participants completed the 10-day program without adverse events, reported high usability, and tolerated immersive exposure well, confirming the safety and acceptability of immersive VR in a hospital setting. Similar improvements in active range of motion, reduced spasticity, and enhanced functional recovery have been previously reported using the same system [26]. The present findings extend this evidence by linking clinical outcomes with detailed kinematic data, providing further insight into the mechanisms underlying motor improvement.

### 4.1. Feasibility and Usability

Immersive VR has repeatedly proven to be a motivating, safe, and well-tolerated rehabilitation adjunct for stroke survivors [42,43,44,45,46,47]. The high System Usability Scale (SUS) scores observed in this study (mean 79 ± 11.3) indicate excellent usability and user satisfaction. Comparable VR-based rehabilitation systems for upper-limb recovery have reported SUS values in the moderate-to-high range (mean ≈ 56–70), supporting the overall feasibility and acceptability of immersive platforms for post-stroke training [31,33,34]. No participant discontinued therapy because of cybersickness or fatigue, further supporting the inclusion of immersive training within structured rehabilitation workflows. The integration of VR into daily sessions proved technically and logistically feasible, requiring minimal setup time and supervision. Such feasibility is crucial for clinical translation, particularly in public rehabilitation units where therapist time and infrastructure are limited.

Positive usability ratings also underscore the role of patient engagement. Middle-aged adults, who constituted the majority of this cohort, typically demonstrate higher digital literacy and greater motivation to engage with technology-based interventions. These psychological and demographic facilitators, combined with the intuitive, game-like design of the platform, likely contributed to strong adherence and overall user satisfaction, both of which are essential for sustained recovery. The positive usability and low simulator sickness scores reinforce the importance of human-centered design in clinical VR systems.

### 4.2. Mechanisms of Motor Improvement

Improvements were most pronounced in the shoulder and elbow joints, suggesting that immersive training primarily enhances voluntary activation and coordination rather than passive flexibility [48,49,50,51]. Because passive range of motion remained stable while active range increased, the observed gains are more consistent with improved voluntary motor recruitment than with changes in joint flexibility. Task-specific, feedback-driven VR training has been shown to increase cortical excitability, promote sensorimotor integration, and facilitate more efficient recruitment of motor units [52,53,54,55].

The observed reduction in the GAP (PROM–AROM) index further supports this notion, indicating greater efficiency in translating available joint range into active movement, a functional marker that has been emphasized in task-oriented post-stroke rehabilitation, where active, not passive, mobility shows the main therapy-related change [56].

Baseline SIAS scores in our cohort (3.4 ± 0.7) indicated mild-to-moderate impairment. This severity range is known to be more amenable to intensive, task-oriented rehabilitation, as SIAS shows good responsiveness and initial motor status is a strong predictor of post-stroke motor recovery [57,58].

From a neurophysiological standpoint, immersive VR engages distributed motor networks through synchronized visual, proprioceptive, and vestibular stimulation, creating goal-oriented, multisensory feedback loops that drive activity-dependent plasticity. Such stimulation enhances functional connectivity among frontoparietal, premotor, and cerebellar circuits, contributing to improved cortical excitability and interhemispheric balance, key determinants of upper-limb recovery [20,27,59].

The closed-loop structure of VR tasks provides continuous real-time error correction and external reinforcement, strengthening the coupling between sensory input and motor output. This process facilitates reweighting of synaptic pathways within the motor cortex and cerebellum, enhancing movement precision and motor recruitment efficiency.

Converging evidence indicates that task-specific, feedback-driven interventions induce structural and functional reorganization across motor and associative regions, including the supplementary motor area, basal ganglia, and parietal cortex. These changes occur through mechanisms of dopaminergic reinforcement and long-term potentiation. [60,61,62,63].

Immersive VR uniquely amplifies these effects by coupling cognitive engagement with sensory-motor reward prediction, thus creating an optimal neurobehavioral context for recovery [64]. Collectively, these findings support the hypothesis that functional improvements observed after VR-assisted training are primarily driven by neuroplastic reorganization rather than passive biomechanical adaptation.

Although the absolute improvements in active range of motion were small (approximately +3° to +5° at the shoulder and elbow), they likely reflect residual neuroplastic potential and enhanced voluntary motor control rather than structural recovery.

Such modest short-term changes are consistent with the subacute/chronic and clinically stable profile of the study cohort and align with previous reports describing comparable magnitudes of improvement following brief, high-intensity hybrid rehabilitation programs.

It should be noted that the short-term changes observed in joint range of motion approach the known measurement uncertainty associated with manual goniometry (±1°). Accordingly, these gains should be interpreted with caution and primarily viewed as indicators of increased neuromotor engagement and voluntary motor control rather than definitive markers of clinically meaningful structural recovery. In this context, the observed improvements likely reflect enhanced motor activation within the available biomechanical range, consistent with early-stage neuroplastic adaptations.

In neurorehabilitation, even small improvements in active joint mobility may hold clinical relevance—particularly in the subacute and chronic stages—by enhancing voluntary control, endurance, and participation in daily activities. Therefore, these gains should be interpreted as indicators of neuromotor engagement and protocol feasibility, rather than as clinically meaningful recovery.

### 4.3. Functional Outcomes and Spasticity Considerations

Although muscle tone was not analyzed statistically, qualitative observations indicated a mild reduction in spasticity, particularly in proximal muscle groups such as the shoulder abductors and elbow flexors, accompanying the observed gains in active range of motion. This pattern suggests that improved voluntary activation and task-specific engagement may have contributed to smoother, less effortful movements, in line with recent evidence that VR-based task-specific training can modulate hypertonia through enhanced reciprocal inhibition [65].

While preliminary, these findings point toward a potential normalization of tone associated with improved motor control, warranting confirmation in future studies using quantitative neurophysiological measures [65].

### 4.4. Individual Variability and Exploratory Digital Performance Metrics

Marked heterogeneity was observed among participants, prompting the use of cluster analysis to better characterize individual response patterns. This exploratory approach identified three response profiles: good, moderate, and poor responders, reflecting the multidimensional nature of post-stroke recovery. The clustering aimed to capture the interaction between clinical improvement and VR performance, allowing differentiation between patients who progressed functionally and those who demonstrated compensatory or plateau responses.

Variability in outcomes revealed that some individuals who showed limited gains in active range of motion nevertheless exhibited consistent improvement in VR performance metrics. Such dissociation indicates that engagement with the immersive environment may enhance visuomotor coordination, cognitive participation, or compensatory motor strategies that are not fully reflected by conventional clinical scales. Conversely, participants with greater AROM improvements tended to display smoother trajectories and higher in-game scores, suggesting partial, but not complete, overlap between biomechanical recovery and digital performance.

The distribution of progress across different VR modules also provided insight into the functional specificity of task design. Each game within the Travee-VR system was tailored to elicit targeted joint movements, such as shoulder abduction and flexion in Rocket and Fires, forearm pronation–supination in Fruits to Basket and Road, or shoulder rotation in Cooking. Participants who engaged more effectively with tasks involving large-amplitude, proximal reaching (e.g., Rocket, Fires) demonstrated greater improvements in shoulder motion, whereas those performing better in fine-motor or distal tasks (e.g., Piano, Diorama) exhibited subtler yet more controlled wrist movements.

This task-specific pattern suggests that each VR module delivered a distinct neuromotor stimulus, reinforcing different aspects of upper-limb control. In several cases, improvement in VR performance occurred even without measurable AROM gains, implying enhanced motor planning, coordination, or sensorimotor anticipation rather than increased joint excursion. Conversely, patients with larger AROM improvements showed smoother, more efficient in-game trajectories, indicating successful transfer of biomechanical gains into functional task execution.

Together, these results support the concept that VR-based exercises can be strategically adapted to target specific movement deficits while simultaneously serving as sensitive, embedded indicators of motor adaptation. In line with the growing field of data-driven neurorehabilitation, these high-frequency, objective performance metrics may represent emerging digital biomarkers of motor learning and neural adaptation. Their integration into adaptive algorithms could enable real-time personalization of task complexity and intensity, providing therapists with continuous quantitative feedback and patients with individualized, dynamically optimized training experiences [66].

Overall, the variability observed across both clinical and digital measures underscores the role of patient engagement, attention, and motivation as key co-determinants of recovery. These cognitive and behavioral dimensions likely influence not only task performance but also the consolidation of neuroplastic changes. Such heterogeneity underscores the need for adaptive, data-driven personalization in VR rehabilitation design.

### 4.5. Cognitive Engagement and Motivational Aspects

The immersive and interactive nature of the Travee-VR platform fostered sustained cognitive engagement throughout the intervention. Attention, motivation, and task-oriented feedback are recognized as key modulators of motor learning and neuroplasticity. During gameplay, participants were continuously exposed to multisensory cues: visual, proprioceptive, and auditory, that reinforced correct movement trajectories and rewarded successful task completion. This immediate, context-rich feedback likely enhanced dopaminergic reinforcement mechanisms, strengthening motor memory consolidation and supporting adherence across the 10-day program.

All participants exhibited preserved cognitive function (MMSE ≥25; mean 27.5 ± 1.4), ensuring adequate comprehension, sustained attention, and active interaction with the virtual environment. Cognitive integrity is essential for meaningful participation in immersive rehabilitation, as deficits in memory, visuospatial orientation, or executive function can limit engagement with feedback-driven tasks. Consequently, pre-treatment cognitive screening using standardized tools such as the MMSE remains crucial for both patient selection and interpretation of treatment variability.

Participants who reported higher subjective enjoyment and presence within the VR environment tended to achieve more stable or accelerated performance improvements, even when clinical gains were moderate. These observations suggest that motivational and emotional engagement may act as amplifiers of neural plasticity, promoting efficient use of residual motor pathways. This pattern is consistent with contemporary neurorehabilitation frameworks emphasizing the integration of cognitive–affective components into motor retraining paradigms [67].

The dynamic challenge–reward balance embedded in the VR scenarios appeared to facilitate adaptive learning. Tasks were neither monotonous nor excessively demanding, fostering a “flow-like” state that maintained engagement and minimized frustration. Such psychological conditions are known to optimize cortical activation, particularly within prefrontal and parietal regions involved in attention, planning, and motor imagery. In this sense, immersive VR provided not only physical but also cognitive training, potentially enhancing executive function, visuospatial processing, and body representation [68].

From a clinical perspective, cognitive engagement may represent more than a secondary benefit, it constitutes a key determinant of long-term rehabilitation success through improved adherence and motivation. Future protocols integrating validated measures of presence, workload, and intrinsic motivation (e.g., NASA-TLX) could better delineate the relationship between engagement and neural recovery. Combining immersive VR with cognitive or dual-task paradigms may further extend these benefits to broader domains of functional independence and quality of life [69].

### 4.6. Translational Implications

The findings of this pilot study highlight the feasibility of integrating immersive VR into conventional neurorehabilitation programs. The Travee-VR platform proved adaptable to standard inpatient workflows, requiring minimal additional resources or therapist supervision. Such ease of integration is critical for clinical adoption, particularly within public healthcare systems where time and personnel are often limited.

Beyond its therapeutic potential, immersive VR offers a novel framework for data-informed rehabilitation. The continuous capture of kinematic and performance metrics enables objective monitoring of patient progress and real-time adjustment of task difficulty. This feature aligns with current trends toward precision rehabilitation, where interventions are individualized based on measurable physiological and behavioral responses [16].

Importantly, the combination of conventional therapy and immersive VR does not aim to replace therapist-guided interventions but to complement them: enhancing engagement, providing consistent feedback, and facilitating quantitative assessment. The integration of such systems could support clinicians in tailoring rehabilitation intensity and tracking recovery trajectories more accurately over time.

The translational value of these approaches extends beyond motor outcomes. By incorporating adaptive algorithms and predictive analytics, future VR-based platforms could identify early markers of recovery potential and optimize therapy planning. These capabilities may also foster continuity of care, allowing transition from supervised clinical sessions to home-based training with remote data monitoring. By enhancing cost-effectiveness and scalability, these frameworks could facilitate the widespread adoption of immersive VR within standard rehabilitation pathways.

Overall, the results support the clinical feasibility and potential scalability of immersive VR-assisted rehabilitation as an adjunctive tool for personalized, feedback-driven stroke recovery.

### 4.7. Limitations and Future Directions

This study has several limitations inherent to its pilot and exploratory design. The small sample size (*n* = 14) and absence of a control group preclude definitive conclusions regarding efficacy and generalizability. Nevertheless, these parameters were adequate for assessing feasibility, safety, and short-term responsiveness, the primary objectives of a proof-of-concept investigation. The heterogeneity of stroke chronicity and baseline motor severity may have introduced interindividual variability; however, this variability also reflects real-world clinical diversity, thereby supporting the external validity of the findings. The cognitive inclusion threshold (MMSE ≥ 25) may have selectively favored individuals with preserved cognitive function, potentially limiting applicability to populations with cognitive deficits. As a pilot investigation, these results should therefore be interpreted as exploratory rather than confirmatory. A limitation of this pilot design is that only general cognitive screening (MMSE) was applied. Nevertheless, future studies should incorporate more comprehensive cognitive assessments beyond the MMSE, focusing on domains such as executive function, visuospatial attention, and working memory, to better elucidate cognitive contributions to variability in motor recovery.

Although lesion side (right, left, or bilateral) was documented for all participants, the limited sample size precluded meaningful subgroup comparisons. No consistent differences in motor improvement were observed between left- and right-hemispheric cases, which aligns with previous evidence suggesting that hemispheric lateralization primarily affects cognitive–perceptual processes rather than pure motor responsiveness. Future studies with larger and more heterogeneous cohorts should investigate how lesion lateralization, neglect, or language deficits may modulate the efficacy and engagement with immersive VR-based rehabilitation.

The statistical approach was intentionally conservative, focusing on within-subject change and non-parametric analyses appropriate for small-sample, non-normal data. Future studies should include larger, controlled cohorts and standardized functional assessments such as the Fugl–Meyer to enable cross-study comparability. Power analysis and confidence interval reporting will strengthen inference robustness, while advanced modeling of kinematic metrics could further elucidate the neural and biomechanical correlates of recovery.

Incorporating adaptive algorithms and multimodal outcome measures, such as electromyography, kinematic profiling, and indices of affect or motivation, could further advance personalization and elucidate the multidimensional effects of immersive VR on recovery. Another important direction is the validation of a clinic-to-home rehabilitation model, enabling autonomous, remotely monitored training. This process has already been initiated, with patients currently participating in home-based Travee-VR sessions as part of an ongoing extension of the program. The resulting data will inform future analyses focused on feasibility, adherence, and long-term functional retention.

Integrating immersive VR within hybrid clinic-to-home rehabilitation frameworks aligns with the growing emphasis on continuity of care and remote, data-supported monitoring. In addition to immersive VR, future research may explore augmented reality (AR)–based rehabilitation paradigms as a complementary or alternative approach. AR systems may offer a less immersive but more accessible and potentially cost-effective solution, particularly for home-based rehabilitation, while still enabling task-oriented feedback and functional motor training in real-world contexts.

Ultimately, this study should be viewed as a proof-of-concept investigation supporting the integration of immersive VR into conventional stroke rehabilitation and paving the way toward scalable, data-driven neurorehabilitation strategies.

The integration of artificial intelligence and adaptive algorithms may further enhance the interpretability of VR-derived data, allowing automated detection of progress or compensatory movement patterns.

## 5. Conclusions

This pilot study supports the feasibility and short-term benefits of integrating immersive virtual reality into post-stroke upper-limb rehabilitation. The Travee-VR system, when combined with conventional therapy, was well tolerated, highly usable, and associated with measurable improvements in active motor performance.

By linking clinical outcomes with detailed kinematic metrics, this study provides preliminary evidence that immersive, feedback-driven environments can enhance voluntary motor control through neuroplastic mechanisms rather than passive biomechanical change.

Importantly, these findings lay the groundwork for the development of a clinic-to-home VR rehabilitation model, extending therapy continuity and patient engagement beyond the hospital setting.

In line with the ongoing shift toward precision and personalized rehabilitation medicine, such data-informed, adaptive systems may enable real-time adjustment of task difficulty and intensity, tailoring therapy to each patient’s unique neurophysiological and behavioral profile. Future large-scale controlled studies are warranted to validate these promising directions and to determine whether the in-game performance metrics identified here can serve as exploratory digital performance metrics of motor responsiveness and recovery potential.

These findings pave the way toward evidence-based integration of immersive VR within standardized stroke rehabilitation protocols.

## Figures and Tables

**Table 1:** Travee-VR Games, Representative Performance Metrics, and Targeted Movements.

Game	Representative Metric Used in Analysis	Targeted Movements/Functional Focus	Game Objective
Fires	Targets extinguished	Shoulder abduction and flexion; proximal reach; visual–motor coordination	Extinguish virtual fires using upper-limb reaching movements
Rocket	In-game score (points)	Shoulder abduction and flexion; rapid activation and motor control	Control a rocket to intercept targets using shoulder elevation
Road	Checkpoints reached	Forearm pronation–supination; bilateral coordination; sustained control	Steer along a virtual road using forearm rotation
Fruits to Basket	Fruits caught	Elbow flexion–extension; forearm pronation–supination; grasp–release coordination	Catch and place fruits into baskets using coordinated reaching
Diorama	Completion rate (%)	Wrist flexion–extension; precision and fine-motor control	Interact with small virtual objects requiring fine control
Cooking	Participation only (qualitative)	Shoulder internal/external rotation; visuomotor planning	Simulate cooking gestures to train shoulder rotation
Piano	Participation only (qualitative)	Wrist flexion–extension; rhythmic and timing coordination	Press virtual piano keys using distal wrist/finger movement

**Table 2:** Demographic and clinical characteristics of patients (N = 14). SIAS U/L = Stroke Impairment Assessment Set, Upper Limb (Proximal).

Sex/Age	No.	Stroke Type (Ischemic/Hemorrhagic)	Lesion Location	Affected Side (Left/Right)	Time from Onset (Months)	SIAS U/L Proximal
M/54	1	Hemorrhagic	Right hemispheric hemorrhage	Left	8 months	4
M/53	2	Ischemic	Right MCA territory infarct	Left	18 months	3
M/57	3	Ischemic	Right vertebrobasilar territory infarct	Left	5 months	4
M/55	4	Ischemic	Left vertebrobasilar territory infarct	Right	6 months	3
F/53	5	Ischemic	Right MCA territory infarct	Left	48 months	4
M/67	6	Ischemic	Right MCA territory infarct	Left	2 months	2
F/73	7	Ischemic	Right MCA territory infarct	Left	18 months	3
M/52	8	Ischemic	Right vertebrobasilar territory infarct	Left	2 months	4
M/48	9	Hemorrhagic	Right hemispheric hemorrhage	Right	2 months	4
M/43	10	Ischemic	Bilateral MCA territory infarct	Bilateral	4 months	4
M/66	11	Ischemic	Right MCA territory infarct	Left	10 months	3
M/58	12	Ischemic	Right MCA territory infarct	Left	48 months	3
M/50	13	Ischemic	Right MCA territory infarct	Left	14 months	2
M/45	14	Hemorrhagic	Right hemispheric hemorrhage	Left	120 months	4

**Table 3:** Feasibility and usability outcomes. Values are expressed as mean ± SD, median, and interquartile range (IQR). SUS = System Usability Scale (0–100; higher scores indicate better usability). SSQ = Simulator Sickness Questionnaire (higher scores indicate greater cybersickness). *p*-values were calculated using the Wilcoxon signed-rank test (significance set at *p* < 0.05). ns = not significant.

Outcome	Mean ± SD	Median	IQR	*p*-Value
SUS_total	79.0 ± 11.3	80.5	17.8	–
SSQ_pre	26.4 ± 11.6	24.3	16.8	–
SSQ_post	35.5 ± 14.4	37.3	22.3	–
SSQ (Δ pre vs. post)	–	–	–	*p* = 0.135

**Table 4:** Baseline (Pre) and post-intervention (Post) clinical outcomes (AROM, PROM, and GAP). Values are mean ± SD (median, IQR). AROM = Active Range of Motion; PROM = Passive Range of Motion; GAP = PROM − AROM. *p* < 0.05 values are indicated in bold.

Outcome	Pre (mean ± SD, Median, IQR)	Post (mean ± SD, Median, IQR)	Δ (Post–Pre)	N Paired	*p*-Value (Wilcoxon)
Shoulder abduction (AROM)	98.6 ± 19.0 (median 100.0, IQR 86.2–110.0)	102.3 ± 18.3 (median 102.0, IQR 90.0–113.8)	3.7 ± 3.7 (median 5.0, IQR 0.0–5.0)	14	**0.0097**
Shoulder abduction (PROM)	120.6 ± 17.0 (median 120.0, IQR 109.2–136.8)	124.8 ± 15.4 (median 123.5, IQR 110.8–137.5)	4.1 ± 4.1 (median 4.0, IQR 0.0–5.0)	14	**0.0074**
Shoulder abduction GAP	22.1 ± 11.2 (median 20.0, IQR 16.2–24.0)	22.5 ± 11.2 (median 20.0, IQR 19.2–22.2)	0.4 ± 3.9 (median 0.0, IQR 0.0–0.0)	14	0.6845
Shoulder flexion (AROM)	101.1 ± 31.9 (median 110.0, IQR 83.2–116.8)	104.4 ± 30.9 (median 110.0, IQR 90.0–121.0)	3.4 ± 3.0 (median 4.0, IQR 0.0–5.0)	14	**0.0065**
Shoulder external rotation (AROM)	41.1 ± 28.1 (median 40.0, IQR 15.8–67.8)	45.3 ± 26.3 (median 42.5, IQR 21.5–69.5)	4.2 ± 3.3 (median 5.0, IQR 1.0–5.0)	14	**0.0045**
Elbow flexion (AROM)	135.3 ± 16.2 (median 140.0, IQR 132.5–143.8)	137.6 ± 14.8 (median 141.5, IQR 134.8–145.8)	2.4 ± 2.6 (median 2.0, IQR 1.0–2.8)	14	**0.0020**
Elbow flexion (PROM)	137.4 ± 14.9 (median 140.0, IQR 136.2–147.2)	141.1 ± 10.9 (median 142.5, IQR 140.0–149.0)	3.7 ± 5.4 (median 2.0, IQR 1.0–4.5)	14	**0.0032**
Elbow flexion GAP	2.1 ± 3.2 (median 0.0, IQR 0.0–5.0)	3.5 ± 7.5 (median 0.0, IQR 0.0–4.8)	1.4 ± 4.8 (median 0.0, IQR 0.0–0.0)	14	0.2850
Wrist extension (AROM)	32.9 ± 22.3 (median 40.0, IQR 11.2–48.8)	35.2 ± 21.4 (median 40.0, IQR 16.2–49.2)	2.3 ± 3.1 (median 1.0, IQR 0.0–3.0)	14	**0.0176**
Wrist extension (PROM)	54.4 ± 18.2 (median 60.0, IQR 50.0–65.8)	56.3 ± 17.1 (median 60.0, IQR 50.0–67.8)	1.9 ± 2.2 (median 1.0, IQR 0.0–4.5)	14	**0.0158**
Wrist extension GAP	21.4 ± 18.4 (median 15.0, IQR 10.0–23.8)	21.1 ± 18.5 (median 14.0, IQR 10.0–22.5)	-0.4 ± 2.9 (median 0.0, IQR 1.8–0.0)	14	0.6049
Forearm pronation (AROM)	80.1 ± 3.5 (median 80.0, IQR 80.0–80.0)	80.3 ± 3.6 (median 80.0, IQR 80.0–80.0)	0.2 ± 0.8 (median 0.0, IQR 0.0–0.0)	14	0.3173
Forearm pronation (PROM)	90.0 ± 0.0 (median 90.0, IQR 90.0–90.0)	90.0 ± 0.0 (median 90.0, IQR 90.0–90.0)	0.0 ± 0.0 (median 0.0, IQR 0.0–0.0)	14	1.0000
Forearm pronation GAP	9.9 ± 3.5 (median 10.0, IQR 10.0–10.0)	9.7 ± 3.6 (median 10.0, IQR 10.0–10.0)	-0.2 ± 0.8 (median 0.0, IQR 0.0–0.0)	14	0.3173

**Table 5:** VR performance metrics (Travee platform). Values are mean ± SD and median [IQR]. *p*-values from Wilcoxon signed-rank test, significance set at p < 0.05. ns = not significant; — = not available. *p* < 0.05 values are indicated in bold.

Game	Metric	First 3 Sessions	Last 3 Sessions	Δ (Mean)	*p*-Value
Fire	Targets extinguished	49.6 ± 39.8, 41.4 [37.4–50.8]	86.1 ± 54.0, 89.3 [45.0–125.5]	36.4	**0.004**
Rocket	Score (points)	35.9 ± 33.0, 25.0 [20.0–31.0]	29.9 ± 13.4, 31.0 [23.0–36.5]	−6.0	0.310 ns
Road	Checkpoints reached	86.4 ± 19.0, 98.3 [78.2–99.6]	89.8 ± 29.9, 100.0 [99.7–100.0]	3.4	0.161 ns
Fruits	Fruits caught	8.7 ± 5.9, 10.0 [4.7–10.5]	15.6 ± 9.6, 13.0 [10.0–20.0]	6.8	**0.034**
Diorama	Completion (%)	12.6 ± 4.2, 14.0 [14.0–14.0]	12.6 ± 4.2, 14.0 [14.0–14.0]	0.0	—
Piano	Played (Y/N)	—	—	Participated	—
Cooking	Played (Y/N)	—	—	Participated	—

**Table 6:** VR responders (top 3 patients by composite score). Δ values represent the difference between the last three and the first three VR sessions. Composite VR score was computed as the mean standardized change across available games (eligibility ≥ 3 games with quantitative metrics). “—” indicates no available data.

Patient ID	Fire Δ (Targets)	Rocket Δ (Points)	Road Δ (Checkpoints)	Fruits Δ (Items)	Diorama Δ (Pieces)	Composite VR Score
4	55	0	55.6	18	—	0.93
3	123.3	17.6	—	—	0	0.87
5	106	28	25	11	0	0.74

**Table 7:** VR-clinical correlations Note: Δ = post–pre difference (last 3 vs. first 3 sessions). Spearman’s ρ and corresponding *p*-values are reported. Only physiologically relevant pairings were tested. Significance set at *p* < 0.05. *p* < 0.05 values are indicated in bold.

VR Game	Clinical Outcome (Δ AROM)	ρ (Spearman)	*p*-Value	N
Fire	Shoulder abduction	0.45	**0.041**	14
Rocket	Shoulder abduction	0.28	0.19	14
Rocket	Shoulder flexion	0.22	0.28	14
Road	Forearm pronation	0.05	0.82	13
Road	Forearm supination	0.11	0.65	13
Fruits	Forearm pronation	0.18	0.39	13
Fruits	Forearm supination	0.09	0.71	13
Diorama	Wrist extension	0.14	0.55	12

## Data Availability

Data provided upon reasonable request.
